# The TAK-003 story: key decisions that shaped development of a tetravalent dengue vaccine

**DOI:** 10.1038/s41541-025-01366-9

**Published:** 2026-02-02

**Authors:** Ian Escudero, Dieter Gniel, Shibadas Biswal, Eckhardt Petri, Gonzalo Perez, Mayuri Sharma, John Weil, Derek Wallace

**Affiliations:** 1 Takeda Vaccines, Inc., Boston, MA USA; 2 Takeda Pharmaceuticals International AG, Zurich, Switzerland

**Keywords:** Live attenuated vaccines, Viral infection

## Abstract

The approved, live-attenuated tetravalent dengue vaccine TAK-003 has undergone extensive preclinical and clinical assessment. This review provides an overview of the rationale for some key decisions made during TAK-003 development. These include decisions around vaccine composition, assessment of factors that may be associated with the safety of live-attenuated viral vaccines, dosing schedule, immunogenicity assessments, and design of the pivotal phase 3 efficacy study.

## Introduction

Dengue is a mosquito-borne disease caused by infection with dengue virus (DENV), of the *Flaviviridae* family, which includes Japanese encephalitis virus, West Nile virus, and yellow fever (YF) virus^1^. DENV is a positive-sense, single-stranded RNA virus comprising 4 antigenically distinct, but closely related, viral serotypes (DENV-1, DENV-2, DENV-3, and DENV-4)^1^. The mature 50-nm diameter virion contains multiple copies of 3 structural proteins, a host-derived membrane bilayer, and a single copy of the RNA genome. The genome is cleaved to form the 3 structural proteins: capsid (C), membrane (M) (with a precursor membrane [PrM] protein) and envelope (E), as well as 7 nonstructural (NS) proteins (NS1, NS2A, NS2B, NS3, NS4A, NS4B, and NS5)^1^.

DENV is transmitted to humans through the bite of an infected *Aedes* mosquito, primarily by female *Aedes aegypti* and *Aedes albopictus* mosquitoes^2^. An estimated 390 million DENV infections occur every year^3,4^, putting a high burden on health care services and causing ~40,500 deaths globally^5–8^. From January to 30 April 2024, over 7.6 million dengue cases were reported in 2024, including 16,000 severe cases and >3000 deaths^9^. However, the true burden of dengue is unknown due to under-reporting and misdiagnosis^4^. The global incidence of dengue has increased 30-fold over the last 50 years^1^, with a rising number of countries reporting their first outbreaks of the disease^4,10^. The increase in dengue cases is due to a number of factors, including failure to eradicate the mosquito vector, urbanization, population growth, the adaptation of mosquito vectors to new environments in response to climate change, and increased travel^4,11–14^. In addition, dengue is a significant risk amongst international travelers, representing the most common infection in returning travelers to the UK^15^. The most frequent regions of acquisition are Southeast Asia, South-Central Asia, and the Caribbean^16^. Whilst dengue has historically been considered a self-limiting disease, it has more recently been associated with potential longer-term complications^17^. As dengue is considered a major threat to global health, the World Health Organization (WHO) is leading an integrated, multi-pronged approach to try to reduce dengue-associated mortality and morbidity^18^. After clean running water, vaccination is the most effective public intervention for disease prevention^19^, and there is an urgent unmet need for a vaccine as part of an integrated dengue control program. Efforts have been made to develop an effective dengue vaccine for the last 75 years; however, numerous challenges have been encountered.

**Fig. 1:**
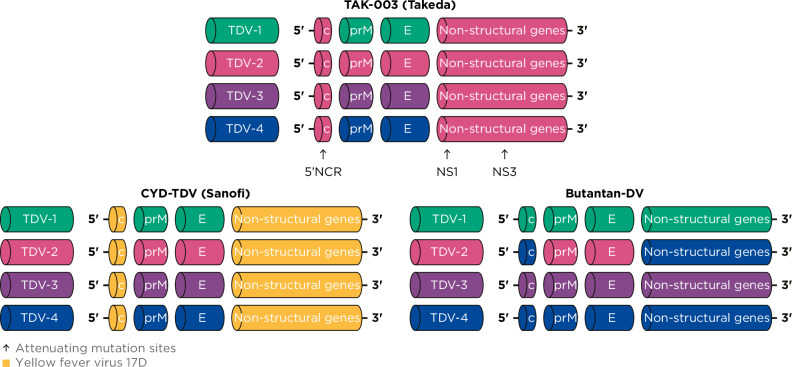
Genetic structure of the TAK-003, CYD-TDV, and Butantan-DV vaccine strains. *C* capsid, *DV* dengue vaccine, *E* envelope, *NS* nonstructural protein, *prM* pre-membrane, *TDV 1/2/3/4* dengue serotype 1/2/3/4 strain.

The first licensed tetravalent dengue vaccine, CYD-TDV (Dengvaxia®, Sanofi Pasteur, Lyon, France), is a live-attenuated vaccine (LAV) consisting of the NS genes of the YF 17D vaccine strain combined with structural prM and E genes of the 4 DENV serotypes (Fig. 1)^20^. CYD-TDV was first approved in 2015, based on data from 2 phase 3 trials with limited assessment of safety and efficacy by baseline dengue serostatus. Initial analysis indicated increased rates of severe and hospitalized dengue in the youngest vaccinees compared with their unvaccinated counterparts^20–22^. Subsequently, a retrospective re-analysis of pooled data from 3 phase 3 CYD-TDV studies demonstrated that, in the absence of previous dengue exposure, the CYD-TDV vaccine increased the risk of severe dengue following breakthrough dengue infection^23^. As a result, use of the CYD-TDV vaccine is restricted to individuals 9–45 years of age (dependent on the transmission intensity of the vaccinating country) who have experienced a previous dengue infection and who live in dengue-endemic regions. CYD-TDV vaccine recipients require serological testing to confirm prior dengue infection before inoculation^20,24^. Sanofi subsequently announced a halt in production of CYD-TDV in 2024 due to low uptake likely because of the need for pre-vaccination screening. The need remains for a vaccine that is well tolerated and efficacious and can be used regardless of recipients’ previous dengue exposure.

An ideal vaccine would be usable in both an endemic setting and in travelers, provide long-lasting protection against all 4 genetically related but antigenically distinct serotypes across a broad age range, and be well tolerated, regardless of serostatus and age at vaccination. Trials should also monitor for vaccine-related environmental risks, including vaccine virus reversion risk, transmission potential of the vaccine virus via the mosquito vector, and risk of vaccine-generated illness due to under-attenuation. Designing a development program to assess the safety and efficacy of a dengue vaccine requires several strategic choices. The aim of this manuscript is to share the scope of and rationale for the choices made during the development of TAK-003.

### TAK-003 overview

Takeda’s live-attenuated tetravalent dengue vaccine, TAK-003, has been investigated in a substantial number of preclinical studies and in a comprehensive clinical development program including 5 phase 1 studies, 6 phase 2 studies, and 8 phase 3 studies (some ongoing). This program was conducted in >28,000 volunteers in 13 countries in both dengue non-endemic and endemic areas, including participants from 18 months up to 60 years of age. These studies assessed the safety, tolerability, reactogenicity, immunogenicity, and efficacy of the tetravalent TAK-003. TAK-003 is currently approved, under the brand name Qdenga (Takeda GmbH, Konstanz, Germany) for use in the European Union^25^, the UK, and several dengue-endemic countries in Latin America and Asia. Regulatory filings are ongoing in a number of additional dengue-endemic countries and phase 4 studies are in preparation.

### Vaccine type and composition

Experience with other flaviviruses found that using an LAV approach was successful for YF and Japanese encephalitis (JE) viruses^26^. Therefore, a live-attenuated approach was selected for TAK-003. LAVs mimic natural infections, generating strong immune responses that can last for decades^26^. However, the principal challenge with LAV viruses is achieving an appropriate level of attenuation; over-attenuation will reduce their immunogenicity and under-attenuation may result in symptomatic manifestations of the disease^26,27^. LAV viruses also have the potential to revert to a wild-type virus. These characteristics all require careful assessment^26^.

Development of TAK-003 began with the clinical assessment of monovalent attenuated versions of DENV-1, DENV-2 (parental virus isolated in Thailand), DENV-3 (parental virus isolated in the Philippines), and -4 (parental virus isolated in Indonesia) in the 1980s in Thailand^28–30^; these were shown to be immunogenic and well-tolerated^29–32^. A tetravalent vaccine was initially assembled by the Centers for Disease Control and Prevention at the Laboratory for Vector-borne Disease based on DENV-1, DENV-2, and DENV-4 components attenuated using serial passaging of wild-type virus in primary dog kidney [PDK] cells and a DENV-3 component serially passaged in monkey kidney cells from Mahidol University and the University of Hawaii^29^. However, the tetravalent vaccine formulation containing the attenuated DENV-2 strain and the 3 other live-attenuated DENV strains was associated with dengue-like clinical symptoms such as headache, rash, and neutropenia. These symptoms were linked with high levels of viremia of the DENV-3 component, which suggested under-attenuation, and that a combination of all 4 attenuated strains was associated with increased reactogenicity^33,34^. In order to reduce reactogenicity and as low levels of viremia were considered a desired characteristic of the vaccine during early development^35^, the live-attenuated DENV-2 strain was chosen as the most promising backbone candidate for the vaccine. The live-attenuated DENV-2 component elicited high rates of seroconversion in seronegative human volunteers, with minimal signs or symptoms of dengue, displaying a good balance between immunogenicity and reactogenicity^31^. This was combined with chimeric DENV-1, DENV-3, and DENV-4 viruses created by replacing the prM and E genes of the DENV-2 with those from wild-type DENV-1, DENV-3, and DENV-4 strains to create the tetravalent construct (Fig. 1)^36^.

Later, DENV-2 was found to be commonly associated with severe dengue disease following secondary dengue infections^37–43^. A recent study based on 19 years of data from Managua, Nicaragua found that severe disease was more prevalent among secondary DENV-2 and DENV-4 cases, while similar disease severity was observed in both primary and secondary DENV-1 and DENV-3 cases^43^. In addition, DENV-2 infection was credited with the highest pooled mortality (2%) in dengue outbreaks reported between 1990 and 2016^44^.

### Assessment of safety concerns potentially associated with the use of LAV vaccines

#### Degree of vaccine virus attenuation

Ensuring that a LAV virus is not over- or under-attenuated is critical in vaccine candidate selection. It remains an important consideration during development as the progression from phase 1 to 3 exposes more trial participants to the vaccine. Comprehensive assessments were conducted throughout the development of TAK-003 to evaluate whether vaccination could lead to a dengue-like syndrome in vaccinees. Phase 1 and 2 studies assessed the presence of viremia and the association with viral-syndrome-like adverse events (AEs), such as fever, headache, arthralgia, and myalgia. In the large-scale phase 3 efficacy study, the focus moved to assessing any relationship between fever and vaccine viremia, with participants in whom fever was identified within 28 days of vaccination sampled to test for viremia. These investigations indicated that while viremia may be associated with some dengue-like systemic symptoms, such as headache or arthralgia, these can also occur in the absence of viremia, illustrating the nonspecific nature of such events^45^. Adequate assessment of risk requires AE clusters to be defined in addition to isolated AEs that are typically assessed in vaccine studies. Rates of fever, rash, or malaise may not suggest a dengue-like syndrome if considered in isolation from each other; however, if they occur in the same individual, they may be indicative of mild dengue disease due to vaccination. Blood samples were taken at multiple intervals after vaccination to assess the potential for thrombocytopenia, neutropenia, and liver function abnormalities that may be associated with dengue infection. These assessments indicated that TAK-003 was not associated with dengue-like syndrome.

#### Risk of reversion of vaccine virus to wild-type

Another important potential concern is vaccine-virus reversion to virulence through mutation. The genetic stability of the DENV-2 PDK-53 backbone has been comprehensively assessed^34^. Three mutations in the DENV-2 PDK-53 backbone attenuate all 4 virus components in the 5′ untranslated region and in the NS protein genes, NS1 and NS3^34,46,47^. These 3 key mutations contribute synergistically and individually to impair the replication of the 4 TAK-003 viruses in vitro, as well as in *Aedes* mosquito cells, and nonhuman primates, and were associated with attenuated neurovirulence in mice^34^. In vitro studies investigating the genetic stability at the 3 attenuation loci found no evidence of reversion for 2 of the 3 attenuation mutations (NS1-53 or NS3-250), with some reversion seen for the 5′NC-57 loci^48^. The individual tetravalent vaccines components are genetically stable during the manufacturing process, ensuring retention of the attenuating mutations^36^.

During assessments of viremia in phase 1–2 studies and the pivotal phase 3 study, replication-competent vaccine virus was detected and sequenced to assess reversion. Reversions were identified in <10% of participants exposed to TAK-003 in phase 1 and 2 studies, and all reversions were at a single attenuation locus, with almost all located in the 5′NCR region. In the pivotal phase 3 study, vaccine viremia occurred infrequently in participants presenting with febrile illness, with low rates of single attenuation loci reversions in those with replication-competent virus. The safety profile in participants with attenuation loci mutations was not indicative of increased symptom severity, with any reported events being nonserious and self-limited in nature^45^. A previous preclinical study has also indicated that reversion of any of the attenuation loci to a phenotype like the wild-type DENV-2 virus required reversions in at least 2 of the 3 loci, with the NS1-53 locus being the most important^46^. This demonstrates that a partially reverted vaccine virus with reversions at a single locus, as seen in rare instances for TAK-003, would still retain the attenuated phenotype.

#### Potential for transmission of the vaccine virus to the environment

An LAV dengue virus may theoretically be transmissible by mosquitoes to the unvaccinated population, with potential safety and/or environmental concerns. Therefore, studies must be performed to assess this risk. Research has demonstrated that each of the individual vaccine components exhibits a lower transmission rate compared with the corresponding wild-type DENV serotype^36^. Replication, infection, transmission, and dissemination of the attenuated DENV-2 PDK-53 virus have been shown to be limited in *Ae. aegypti* and *Ae. albopictus* cells and mosquitoes^46,49–51^. As TAK-003 is a tetravalent vaccine, it was important to confirm transmission potential for the whole vaccine as well as the individual strains. TAK-003 demonstrated incompetent or defective infection and dissemination in *Ae. albopictus* mosquitoes^50^. Taken together, these data suggest that the TAK-003 vaccine virus is unlikely to be transmitted by either the primary or secondary vectors.

### Assessing the complex immune response to dengue and its role in vaccine efficacy

#### The anti-dengue immune response

Dengue infection is complex and can progress to severe disease with little warning. The immune response to dengue virus is multi-pronged, layered, and sequential, involving both innate and adaptive immunity to the first and subsequent dengue exposures. The immune responses driving protection from infection may not necessarily overlap with those driving protection from severity^52^. The immune response is also affected by the infecting dengue serotype and prior dengue or flavivirus exposure. This can potentially lead to intensified forms of disease in individuals, thought to be at least partially due to antibody-dependent enhancement, caused by low levels of pre-existing anti-DENV neutralizing antibodies (NAbs)^53^.

This observation linking NAbs titers to increased severity has resulted in an assumption that a dengue vaccine must generate a balanced, serotype-specific, humoral immune response^52^. As there is a lack of a validated and universal correlate of vaccine protection for use in clinical trials, the primary immunological endpoint has been to induce high anti-dengue NAb titers. However, linking vaccine efficacy to NAb magnitude or specificity alone is an oversimplification of the complex immune response driving protection against infection and disease progression, as well as the role that different elements of the virus structure play in the phases of infection and disease^52^. Potent NAbs make up <5% of antibody responses to flavivirus infections and vaccination^54^, and defining efficacy based only on NAb titers does not take into consideration the effector functions of antibodies that have been shown to play a significant role in neutralization of dengue and prevention of disease progression. These include anti-dengue Fc effector antibodies, such as complement-fixing antibodies, and anti-NS1 antibodies, which can contribute to providing protection against symptomatic dengue infection and symptoms of severe disease^52,55–57^. In addition, effective cell-mediated functions, including memory B- and T-cell responses, are critical for protection against subsequent symptomatic dengue infections following vaccination. These responses represent a key aspect of long-term protection against dengue. Indeed, the increased severity of dengue infection observed in seronegative individuals after CYD-TDV vaccination is hypothesized to be due to the absence of dengue-specific NS proteins (virulence factors) in the vaccine (Fig. 1)^58–60^, which play an important role in cell-mediated immunity^61^. In contrast to CYD-TDV, the dengue backbone of TAK-003 promotes immune responses against these dengue-specific, conserved DENV-2 NS proteins (Fig. 1)^60^.

As different immune responses may contribute to different types of protection, including from infection and disease severity, it is not surprising that vaccine efficacy may not necessarily correlate solely with the presence of NAbs to each of the 4 dengue serotypes. Furthermore, dengue vaccines that have not prevented initial DENV infection may be capable of eliciting a combination of innate and adaptive immunity involved in preventing disease progression.

#### Assessment of the immune response to TAK-003

Based on recommendations on dengue vaccine immuno-characterization^62^, a large variety of immunological endpoints were assessed during the TAK-003 clinical development program using an extensive range of assays (Table 1). TAK-003 was broadly immunogenic against all serotypes, regardless of baseline dengue serostatus, throughout the development program^63–68^. TAK-003 elicits tetravalent NAbs to all 4 dengue serotypes, which consist of a mixture of serotype-specific and cross-reactive antibodies^69–71^. However, relative magnitude and frequency of type-specific antibodies did not necessarily correlate with efficacy by serotype in baseline seronegative vaccine recipients. Early efficacy data from clinical trials assessing the investigational, live-attenuated, tetravalent Butantan-dengue vaccine (DV), which consists of the full-genome DENV-1, DENV-3, and DENV-4 components with a DENV-2/DENV-4 chimeric backbone (Fig. 1)^72^, indicate that this pattern will persist, with lower and declining vaccine efficacy observed against DENV-2, despite observations of similar immunogenicity^21,73,74^.

**Table 1:** Overview of exploratory immunologic assessments of TAK-003

Trial	Population	Trial group, formulation (dosing schedule)	Assay	Sampling days
DEN-102	Adult	Group 1, LD-TDV (Day 0 and Day 90)	Transcriptional profile	0, 2, 4, 7, 90, 92
DEN-203	Pediatric, adolescent, adult	Groups 1–5, HD-TDV (Day 0 and Day 90)	Anti-dengue NS1 IgG ELISA (DENV-1, -2, -3, -4)	0, 120
			Anti-dengue NS1 IgG ELISA (DENV-2 only)	0, 28, 90, 120, 180, 360
			DENV total binding IgG ELISA	0, 120, 180
		Groups 2–5, HD-TDV (Day 0 and Day 90)	DENV-2 depletion and RVP neutralization assay (type-specific neutralizing antibodies)	0, 120
		Groups 1–4, HD-TDV (Day 0 and Day 90)	Anti-dengue IgG avidity assay	0, 28, 90, 120, 180, 360
			Anti-dengue complement antibody assay	0, 28, 90, 120, 180, 360
		Groups 1, 2, 3, and 5, HD-TDV (Day 0 and Day 90)	Endothelial hyperpermeability (trans-endothelial electrical resistance)	0, 120
DEN-204	Pediatric, adolescent	Group 1, TDV (Day 1 and Day 91)	Anti-dengue NS1 IgG ELISA	1, 180
			DENV total binding IgG ELISA	1, 180
			Anti-dengue IgG avidity assay	1, 180
			Anti-dengue complement antibody assay	1, 180, 360
			DENV-2 depletion and RVP neutralization assay (type-specific neutralizing antibodies)	1, 180
		Groups 1–4, Immunogenicity subset, Panama only, TDV (Day 1 and Day 91/Day 1 only/Day 1 and Day 365) or Placebo (Days 1, 91, and 365)	Intracellular cytokine staining	1, 91, 180, 360, 540
DEN-205	Adult	TDV, TDV (Day 1)	Quad-color fluorospot	1, 30, 180
		HD-TDV, HD-TDV (Day 1)	Dengue MNT – breadth of neutralization	1, 30
		TDV, TDV (Day 1)	Dengue MNT – breadth of neutralization	1, 30
		TDV, TDV (Day 1)	IFN-γ ELISpot	1, 30, 180, 365
DEN-301	Pediatric, adolescent	TDV, immunogenicity subset, TDV (Day 1 and Day 90)	Anti-dengue NS1 IgG ELISA	1, 120, 270, 450
			DENV total binding IgG ELISA	1, 120, 270, 450
			Anti-dengue IgG avidity assay	1, 120, 270, 450
			Anti-dengue complement antibody assay	1, 120, 270, 450
			DENV-2 depletion and RVP neutralization assay (type-specific neutralizing antibodies)	1, 120, 270
			DENV 1-4 mutant RVP neutralization assay (cross-neutralization)	1, 365, 365 to 480
DEN-304	Adult	Group 3, TDV Lot 3 (Day 1 and Day 90)	Anti-dengue NS1 IgG ELISA	1, 120, 270
			DENV total binding IgG ELISA	1, 120, 270
			Anti-dengue IgG avidity assay	1, 120, 270
			Anti-dengue complement antibody assay	1, 120, 270
			DENV-2 depletion and RVP neutralization assay (type-specific neutralizing antibodies)	120, 270
DEN-313	Pediatric, adolescent	Immunogenicity subset, TDV (Day 1 and Day 90)	IFN-γ ELISpot	1, 30, 120, 270

*DENV* dengue virus, *ELISA* enzyme-linked immunosorbent assay, *ELISpot* enzyme-linked immunosorbent spot, *HD-TDV* high-dose TDV, *IFN-γ* interferon gamma, *IgG* immunoglobulin G, *LD-TDV* low-dose TDV, *MNT* microneutralization test, *NA* not applicable, *NS1* nonstructural protein 1, *RVP* reporter virus particle, *TDV* tetravalent dengue vaccine.

TAK-003 has also been shown to elicit a durable, DENV-specific, polyfunctional cell-mediated immune response, including activation of both CD8+ and CD4+ T cells, with focused reactivity against NS1, NS3, and NS5 proteins^75–78^. This cell-mediated immunity exhibited significant cross-reactivity against all 4 DENV serotypes^77^. In addition, TAK-003 elicits tetravalent type-specific and cross-reactive memory B cells to all 4 DENV serotypes^79^ as well as production of complement-fixing antibodies against all 4 DENV serotypes. These antibodies persisted for at least 1 year post vaccination, irrespective of baseline serostatus, and correlated with NAb titers, indicating that antibodies produced after TAK-003 vaccination are functional in both complement activation and neutralizing virus infection by all DENV serotypes^80^. In addition, TAK-003 elicited humoral response to the vaccine DENV-2 NS1, which were cross-reactive against NS1 from DENV-1, DENV-3, and DENV-4, and these antibodies were shown to be functional in blocking NS1-mediated endothelial hyperpermeability in vitro, suggesting a potential for reducing severe dengue^56^. Our understanding of DENV immunology and the interplay between humoral and cellular immune responses has advanced in recent years but remains incomplete. Vaccines should, therefore, aim to elicit broad immune responses directed at dengue proteins. TAK-003 engages both innate and adaptive arms of the immune response, and both humoral and CMI-associated adaptive immunity. These broad immune responses provide increased confidence in the efficacy profile and suggest that TAK-003 has the potential to have a considerable global health impact .

**Table 2:** Key decisions made during the TAK-003 clinical development program

Development considerations	Key observations and decisions	References/key studies
Vaccine type and composition	• Live attenuated approach selected • Single DENV-2 backbone with chimeric DENV-1, -3, and -4 components chosen for balance between immunogenicity and reactogenicity	^31,34,36^
Degree of vaccine virus attenuation	• Attenuation achieved via 3 mutations in DENV-2 PDK-53 backbone (5’NC-57, NS1, NS3) • Comprehensive assessment of association between vaccination and occurrence of dengue-like syndrome was performed • Level of attenuation is sufficient and vaccination with TAK-003 is not associated with dengue-like syndrome	^45,46^
Risk of reversion	• Genetic stability thoroughly assessed, no evidence of reversion for 2 of the 3 attenuations mutations, with very low levels of reversion seen for the 5′NC-57 loci • Reversion of any of the attenuation loci to a phenotype like the wild-type DENV-2 virus required reversions in at least 2 of the 3 loci • Risk of reversion is low and even in rare instances of a single locus reversion, the vaccine maintains the attenuated phenotype	^36,45,46,48^
Transmission potential	• Each of the individual vaccine components exhibits a lower transmission rate compared with the corresponding wild-type DENV serotype • TAK-003 and its components demonstrated incompetent or defective replication, infection, transmission, and dissemination in *Ae. Aegypti* and *Ae. Albopictus* mosquitoes • TAK-003 is unlikely to be transmitted by either the primary or secondary vectors	^46,49–51^
Assessing the immune response to TAK-003	• Dengue infection and the associated immune response are complex and there is currently no established correlateofprotection • Use of NAbs alone to indicate vaccine efficacy may not accurately reflect the complex immune response • As a result, a large variety of immunological endpoints were assessed during TAK-003 clinical development	^52–54,63–71,75,76,78^
Viremia, vaccine virus replication, and vaccine safety and efficacy	• Assessment of viremia is often inaccurately conflated with vaccine-virus replication and vaccine efficacy • Viremia does not necessarily correlate with vaccine efficacy, as seen for the DENV-1 component of TAK-003	^45,81^
Dosing schedule	• Higher tetravalent seropositivity rate observed when a second dose of the vaccine was given after 3 months compared with a 1-dose schedule in baseline seronegative individuals • A schedule consisting of a 2-dose regimen, administered at Months 0 and 3, was selected to advance to phase 3 clinical trials	^63–66,83,84^
Pivotal trial study design	• Study was designed in compliance with the WHO guidelines on vaccine development • Multi-country study was chosen to ensure heterogeneity of dengue epidemiology and flavivirus exposure • An age range of 4–16 was chosen to reflect the pathophysiology of dengue to ensure high enough incidence of febrile illness to assess vaccine efficacy • Baseline blood samples taken to allow stratification of data by baseline serostatus	^87^

### The relationship between viremia, vaccine virus replication, and vaccine safety and efficacy

Replication of an LAV virus is an important prerequisite for generation of a robust immune response; however, it is impractical to assess local virus replication in the context of vaccine clinical development. Measurement of viremia in peripheral blood is more straightforward and, as a result, is often inaccurately conflated with replication. The absence of viremia is not necessarily the same as the absence of replication and, therefore, should not be used as the only indicator of potential vaccine efficacy. This is supported by the demonstration of robust efficacy of TAK-003 against DENV-1 (vaccine efficacy of 52.3% [95% CI 42.6–60.3] against virologically confirmed dengue [VCD] and vaccine efficacy of 71.2% [95% CI 51.2–82.9] against hospitalized VCD)^81,82^, despite a lack of detectable viremia for the DENV-1 component measured in the associated clinical trials^67^. As described in the section on safety concerns with the use of LAV vaccines, the presence of viremia is more often associated with potential safety concerns, particularly the occurrence of a dengue-like syndrome including headache, rash, myalgia, and potential neutropenia, thrombocytopenia, or the rise of liver function enzymes.

### Determination of the TAK-003 dosing schedule

Dosing levels and schedules, as well as methods of administration of TAK-003, were studied in numerous phase 1 trials^63–66^. Data demonstrated a higher tetravalent seropositivity rate when a second dose of the vaccine was given after 3 months compared with a 1-dose schedule in individuals who were seronegative at baseline^83,84^. Therefore, a schedule consisting of a 2-dose regimen, administered at Months 0 and 3, was selected to advance to phase 3 clinical trials. This was selected despite the potential compliance challenges associated with a second vaccine dose. The benefit of this 2-dose schedule was confirmed in the pivotal phase 3 study, with tetravalent seropositivity of 85.3% 1 month after the first dose and 99.5% 1 month after the second dose in baseline seronegative participants^85^. A 3-month schedule allowed this improved seroconversion to be achieved rapidly. As many factors contribute to immune protection, seroconversion of a seronegative individual receiving a dengue vaccine cannot be seen as proof of seroprotection; however, the absence of seroconversion to a specific serotype was taken to suggest a potential lack of protection against that serotype. Importantly, the second dose was not intended to improve the immune response in those who had responded to the first dose, but to complete the response to all serotypes at an individual level. This is a common approach in live viral vaccine development and is particularly important for a multivalent vaccine. This would be particularly relevant in the context of public and large-scale vaccination programs. This 2-dose schedule allowed early coverage of individuals who had not previously seroconverted to a particular dengue serotype and rapidly increased the proportion of responders to all 4 serotypes.

### Key design considerations for the pivotal phase 3 efficacy study

The pivotal phase 3 Tetravalent Immunization against Dengue Efficacy Study (TIDES) (NCT02747927) investigated TAK-003 in >20,000 children aged 4 to 16 in 8 dengue-endemic countries in Latin America (Brazil, Colombia, Panama, Dominican Republic, and Nicaragua) and Asia (Philippines, Thailand, and Sri Lanka). The study was designed in compliance with the WHO guidelines on vaccine development.

Sites were chosen based on numerous predefined criteria, including the demonstrated burden of dengue disease, the investigators’ experience, and healthcare pathways and processes. A multi-country study was chosen to ensure heterogeneity of dengue epidemiology (circulating serotypes) and flavivirus exposure (JE/YF vaccination or Zika infection). This enabled the assessment of the efficacy of TAK-003 against all 4 serotypes as well as the impact of flavivirus exposure on the safety and efficacy of the vaccine^82^.

An age range of 4 to 16 reflected the pathophysiology of dengue; primary and secondary infections are far more likely to be symptomatic and, therefore, detected by febrile surveillance, than subsequent infections that tend to be asymptomatic. In a highly endemic setting appropriate for a dengue efficacy study, the inclusion of adults is unlikely to contribute to the assessment of efficacy due to the likely absence of symptomatic dengue fever in individuals who have had multiple prior dengue exposures. To detect a primary or secondary infection in an adult would be possible in a low endemic setting, but the incidence would likely be too low to support an efficacy study. Consider the likelihood of detecting a case of dengue during 5 years of follow-up in a 40-year-old individual who had only been exposed to dengue on ≤1 occasion. However, it is important to note that in recent years the epidemiology of dengue has been changing, particularly in Asia, with the mean age of dengue hemorrhagic fever increasing^86^.

**Fig. 2:**
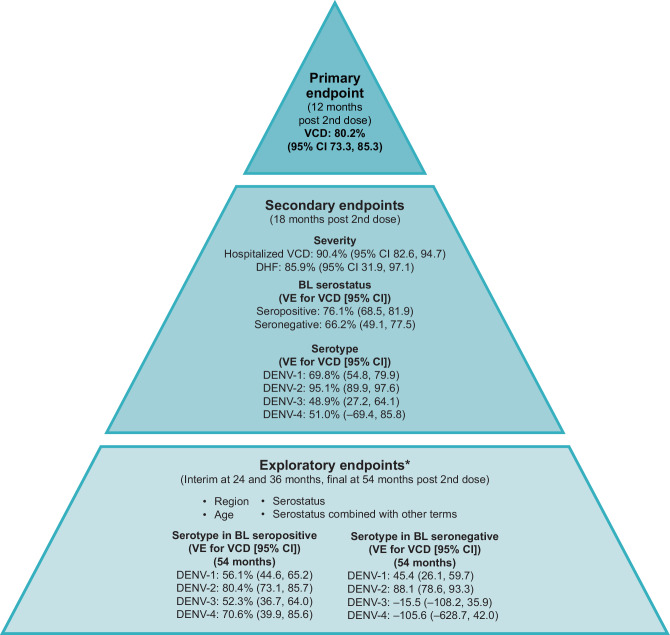
Overview of Tetravalent Immunization against Dengue Efficacy Study (TIDES) efficacy endpoints. Baseline seronegative – baseline reciprocal neutralizing titer of <10 for all 4 dengue serotypes as determined by MNT 50% assay. Baseline seropositive – baseline reciprocal neutralizing titer of ≥10 for at least one dengue serotype as determined by MNT 50% assay. ^*^Not powered to support definitive conclusions due to low number of observations. These analyses are descriptive only. *BL* baseline, *CI* confidence interval, *DENV* dengue virus, *DHF* dengue hemorrhagic fever, *MNT* microneutralization test, *VCD* virologically confirmed dengue, *VE* vaccine efficacy.

The primary endpoint of the study was assessed 12 months after the second dose of TAK-003. Secondary efficacy endpoints included efficacy by serotype, by serostatus, and by severity (hospitalization, dengue hemorrhagic fever, and severe dengue as determined by an independent Dengue Case Adjudication Committee). These were assessed 18 months after the second dose to allow for inclusion of a larger number of dengue cases, to increase the precision of these subgroup analyzes (Fig. 2).

Baseline (prevaccination) blood samples were taken from all participants to ensure that safety and efficacy analyzes could be performed by baseline serostatus. Dengue cases were identified by febrile surveillance supported by weekly contact with all participants throughout 57 months of follow-up and detected by serotype-specific PCR of samples taken within 5 days of fever onset. The collection of samples within 5 days from fever onset was important to ensure the ongoing presence of dengue viremia.

It was critical to ensure individual sites were able to implement febrile surveillance and that samples could be collected irrespective of where a participant presented with disease. Febrile surveillance and healthcare mapping were tested at most sites in a dry run that, in some instances, ran for months before vaccination. The rate of collection of samples within 5 days of fever onset was monitored throughout the study. This rigorous approach led to a viable PCR result being available in 98.1% of fevers detected in the study, 94.4% of which were taken within 5 days of fever onset^87^. This was despite challenges such as the COVID-19 pandemic. It is also worth noting that dengue case management followed local practices and standard-of-care varied across different trial sites. This led to differences in, for example, hospitalization rates, which is a confounding factor and could impact efficacy data for hospitalized VCD.

The above considerations are a few examples of the many complex design and operational considerations necessary for the conduct of a high-quality dengue efficacy study.

## Conclusions

With increasing geographic spread, frequency, and magnitude of outbreaks, dengue continues to pose a major public health threat worldwide. There is an urgent unmet need for a dengue vaccine as part of an integrated, multi-pronged dengue control program. However, due to the complexities of live viral vaccine development, the specific challenges posed by a disease as complex as dengue, and the safety issues identified with the CYD-TDV vaccine, dengue vaccine development is a particularly intricate, long, and expensive endeavor. The aim of this article is to share some of the development considerations specific to TAK-003 and the rationale for some of the key decisions made during its development (Table 2). TAK-003 was designed to elicit a balanced and broad immune response, has undergone extensive investigation to assess environmental and biological safety, and has demonstrated long-term protection against VCD and hospitalized VCD, irrespective of prior dengue exposure. The remaining data gaps from phase 3 development (i.e., the TAK-003 profile against DENV-3 and DENV-4 in baseline seronegative individuals) are planned to be further assessed in a post-authorization effectiveness study. This multi-country, nested case-control study is planning to enroll a cohort of 70,000 participants from high dengue transmission areas with known circulation of DENV-3 and DENV-4 to evaluate the impact of TAK-003 on severe/hospitalized dengue cases due to these serotypes. This study specifically aims to evaluate whether serotype-specific hospitalization (in particular hospitalization due to DENV-3 or DENV-4) is increased or decreased amongst individuals who were dengue seronegative at the time of vaccination compared with unvaccinated seronegative individuals. Following the launch of the vaccine, Takeda is committed to ongoing routine post-marketing pharmacovigilance surveillance to assess any adverse events associated with the vaccine in the real-world setting. TAK-003 will continue to be monitored in different geographies in ongoing and additional post-licensure and real-world studies. The safety and efficacy of a booster dose of TAK-003 are also currently under evaluation. The Strategic Advisory Group of Experts on Immunization states that, although potential risk of severe dengue in seronegative vaccinated individuals following DENV-3 or DENV-4 infection cannot be ruled out based on the available data, TAK-003 be considered for inclusion into existing national immunization programs. This is recommended in high transmission areas, where dengue is a significant public health problem, for individuals 6 to 16 years of age^88^. Overall, TAK-003 has the potential to have a significant impact on the global burden of dengue, as part of a combined, multimodal dengue prevention and control strategy.

## Data Availability

The data sets, including the redacted study protocol, redacted statistical analysis plan, and individual participants' data supporting the results of the completed study, will be made available within 3 months from initial request, to researchers who provide a methodologically sound proposal. The data will be provided after its de-identification, in compliance with applicable privacy laws, data protection and requirements for consent and anonymization. Data requests should follow the process described in the Data Sharing section on https://clinicaltrials.takeda.com/ and https://vivli.org/ourmember/takeda/.
