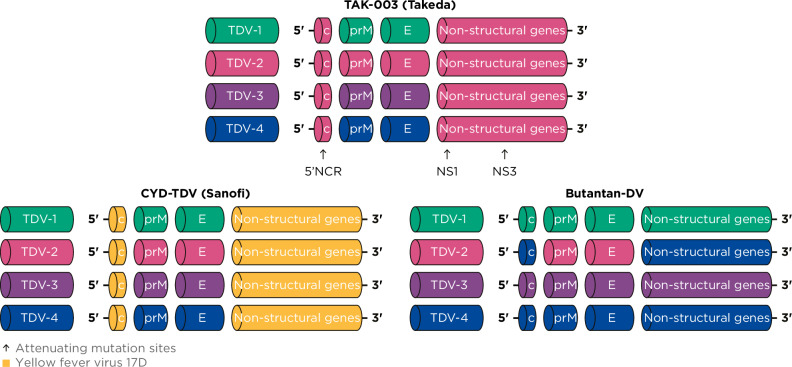# Author Correction: The TAK-003 story: key decisions that shaped development of a tetravalent dengue vaccine

**DOI:** 10.1038/s41541-026-01471-3

**Published:** 2026-05-06

**Authors:** Ian Escudero, Dieter Gniel, Shibadas Biswal, Eckhardt Petri, Gonzalo Perez, Mayuri Sharma, John Weil, Derek Wallace

**Affiliations:** 1 Takeda Vaccines, Inc., Boston, MA USA; 2 Takeda Pharmaceuticals International AG, Zurich, Switzerland

**Keywords:** Live attenuated vaccines, Viral infection

Correction to: *npj Vaccines* 10.1038/s41541-025-01366-9, published online 02 February 2026

In the original Article, Figure 1 contained an error in the Butantan-DV panel. The non-structural (NS) region of the TDV-2 construct was incorrectly coloured yellow (representing yellow fever virus 17D) instead of blue (representing DENV-2). This has now been corrected in the HTML and PDF versions of the Article.

Incorrect Figure 1
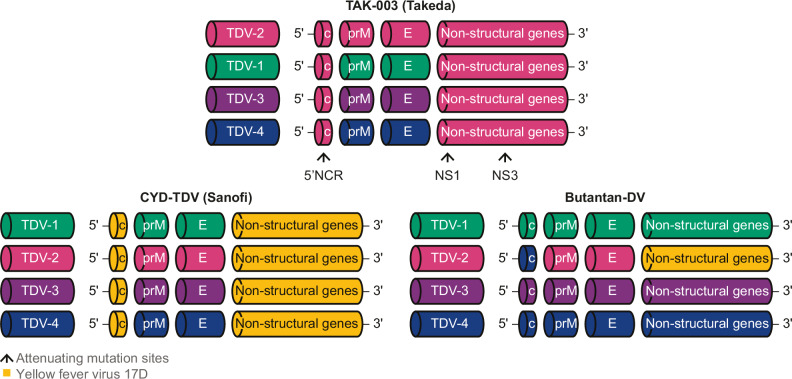


Correct Figure 1